# Prevalence of tobacco smoking among school teachers in Botswana

**DOI:** 10.1186/1617-9625-11-24

**Published:** 2013-11-27

**Authors:** Patience N Erick, Derek R Smith

**Affiliations:** 1 School of Health Sciences, Faculty of Health, University of Newcastle, Ourimbah, New South Wales, Australia

## Abstract

**Background:**

Tobacco is a leading cause of death worldwide, and nearly 80% of all smokers live in low to middle income countries. Previous research has suggested that smoking rates vary by occupation, with relatively low rates commonly seen among educators. Despite this fact, little is known about the smoking habits of teachers in Botswana. The objective of this study, therefore, was to investigate prevalence and correlates of tobacco use among school teachers in Botswana.

**Results:**

The prevalence of smoking among school teachers in Botswana was found to be relatively low. Of the 1732 participants in the study, only 3.2% reported being current smokers, 5.3% were ex-smokers and 91.5% had never smoked. Smoking was more common among male teachers when compared to females, being 10.8% and 0.4%, respectively. Factors such as school level, marital status and body mass index were found to be positively associated with tobacco smoking, whereas age, length of employment and weekly working hours were not.

**Conclusion:**

This study suggests that Botswana school teachers have a low prevalence of tobacco smoking. While this result may be attributed to tobacco control measures that have been put in place, there is still need to put in place systems to monitor compliance and programs to help those who want to quit smoking. Such protocols would represent a major step forward in further reducing the prevalence of smoking in the education profession.

## Introduction

Tobacco use represents one of the most important public health problems worldwide. Tobacco endemic is a leading cause of death, illness and impoverishment, resulting in nearly six million fatalities annually. Over 90% of these deaths are caused directly by tobacco use whilst about 10% are the results of non-smokers being exposed to second-hand smoke [1]. If current trends are not changed, these figures are expected to increase to more than 8 million deaths per year by 2030 [1,2]. Nearly 80% of the more than one billion smokers worldwide, a percentage projected to rise [3], live in low and middle income countries where the burden of tobacco related illness and death is substantial. Premature deaths which may be caused by tobacco use deprive families of those who died of income, raise the cost of health care and hinder economic development [1]. Additionally, tobacco smoking is a prevalent risk factor for cardiovascular and respiratory disease such as coronary heart disease, lung cancer and tuberculosis [1,3]. In a study that was conducted in Botswana, it was found that, 66.4% of patients that were diagnosed and treated for cancer in three referral hospitals were associated with tobacco use [4]. Moreover, tobacco use represents an important issue in occupational health because of its significant impact in the workplace [2]. Previous research has suggested that smoking rates vary by occupation, with relatively low rates commonly seen among educators [5-7], although little is known about the smoking habits of teachers in African countries.

Teachers have an important responsibility in tobacco control given that they are highly respected in their communities as they influence the evolution for each aspect of life [8,9]. It has been recognised that teachers are important role models for students, conveyors of tobacco prevention curricula and key opinion leaders for school tobacco control policies [9,10]. In addition, teachers have daily interaction with students and thus represent an influential group in tobacco smoking control. However, this potential can be limited if teachers use tobacco especially in the presence of students in school premises [10]. The results of a study carried out in Nairobi, Kenya to determine the prevalence and risk factors of smoking among secondary school students indicated that, smoking among students started very early in their life due to the smoking habits of their parents at home and teachers at school [11]. Similar results were found in the study conducted to assess the influence of smoking and tombak (local smokeless tobacco) dipping by parents, teachers and friends on cigarette smoking and tombak dipping by school going Sudanese adolescents [12].

Despite the important role of teachers on tobacco smoking control, few studies have been conducted to investigate tobacco smoking behaviours of school teachers. As far as the authors of this study could ascertain, no study on tobacco smoking has been conducted among teachers in Botswana. The aim of this study was, therefore, to investigate and report on the prevalence of tobacco smoking among teachers in Botswana.

## Methods

As part of a larger descriptive cross sectional study of occupational health issues, 3 100 school teachers in Botswana were surveyed. The study was approved by the University of Newcastle Human Research Ethics Committee and Botswana Ministry of Education and Skills Development. From seven education regions, 107 primary and 57secondary schools were randomly selected. All school teachers in those schools were invited to take part in the study. Permission to conduct the research in the selected schools was sought from school heads. Informed consent of teachers was implied by completing and returning the questionnaire. Data was collected from August to December 2012 by means of an anonymous, self-reporting questionnaire. Tobacco smoking variables were constructed to estimate cigarette smoking prevalence, and proportions of ex-smokers and those who have never smoked. Data was also collected on the number of cigarettes smoked daily and number of years since quitting to smoke. SPSS 20.0 was used to analyse the collected data. Pearson’s chi-square tests were used to determine statistical associations with smoking.

## Results

An overall response rate of 56.3% was obtained in this study. Out of the total respondents 1260 (72.7%) were females, 832 (66.0%) of which were primary school teachers with mean age of 39.34 ± 9.02 years and working experience of 13.36 ± 8.82 years. The results of this study show that 3.2% of school teachers in Botswana reported that they were current smokers, while 5.3% were ex-smokers and 91.5% have never smoked. The results of the current study, as indicated in Table 1, reveal that gender was significantly associated with smoking among school teachers. The prevalence of smoking among female teachers (0.4%) was substantial lower than of their male counterpart (10.8%), p < 0.001. Marital status was significantly associated with tobacco smoking (p = 0.001). School level has also been positively associated with tobacco smoking among teachers. Majority of smokers were 30 years or less. Age and length of employment were not significantly associated with tobacco smoking.

**Table 1:** Prevalence of tobacco smoking among teachers in Botswana

	**Current smoker**	**Ex-smoker**	**Never smoked**	**p-value**
	**n (%)**	**n (%)**	**n (%)**	
Total	56 (3.2)	91 (5.3)	1585 (91.5)	
**Gender**				**<0.001**
Male	51 (10.8)	64 (13.6)	357 (74.6)	
Female	5 (0.4)	27 (2.1)	1228 (97.5)	
**Age range (years)**				0.172
≤30	19 (5.3)	19 (5.3)	319 (89.4)	
31-40	22 (3.4)	35 (5.4)	591 (91.2)	
41-50	12 (2.3)	28 (5.3)	485 (92.4)	
>50	2 (3.2)	7 (4.3)	154 (94.5)	
**School level**				**0.002**
Primary school	26 (2.6)	40 (4.0)	937 (93.4)	
Junior secondary	27 (4.8)	36 (6.4)	493 (88.7)	
Senior secondary	3 (1.8)	15 (8.8)	152 (89.4)	
**Marital status**				**0.001**
Single	42 (4.6)	49 (5.3)	827 (90.1)	
Married	8 (1.1)	39 (5.5)	666 (93.4)	
Separated/divorced/widowed	6 (5.9)	3 (3.0)	92 (91.1)	

## Discussion

About 3.2% of teachers in this study reported that they were smokers. This prevalence is relatively lower compared to results of other studies that have been carried out around the world. Supporting this are the results of studies from Kingdom of Bahrain and Kenya in which prevalence of smoking among Bahraini and Kenyan teachers were 7% for each [13,14]. As shown on Table 2, quite similar findings were found in studies conducted among Malay and Yemen teachers where 7.8% and 8% prevalence were reported, respectively [9]. Similarly high prevalence of tobacco smoking has been reported among school teachers around the world. A study of school teachers in India, for example, found that 14.5% of primary school teachers where smokers [15] while in Bangladesh prevalence of tobacco smoking among secondary school teachers was 17% [16] and 17.8% in Sousse, Tunisia [17]. Furthermore, another study of Malay secondary school teachers in Kelantan found that 20% are smokers [18]. A much higher prevalence was reported in a study from Tunisian Sahel which found that 29.3% of school teachers smoked [19] and 29.7% of primary and secondary Spanish teachers were smokers [20]. The highest smoking prevalence (58.1%) has been reported by Turkish primary teachers. In the same study, 36.1% teachers reported that they were ex-smokers whilst 5.8% had never smoked [21]. A similar smoking prevalence (52.1%) was reported among Syrian male primary and secondary school teachers [22], whilst in Malaysia, 40.6% secondary school teachers were smokers [23].

**Table 2:** Prevalence of smoking among school teachers reported from international studies

**Country**	**Participants**	**Number of participants**	**Response rate (%)**^**a**^	**Smokers (%)**	**Ex-smokers (%)**	**Never smoked (%)**	**Year**^**b**^	**Authors**
India	Primary school teachers		400*	14.5			2013	Savadi *et al.*[15]
Malaysia	Secondary school teachers		495*	7.8			2012	Al-Naggar *et al.*[9]
Tunisia		800	92.4	17.8			2011	Harrabi *et al.*[17]
Bangladesh	Secondary school teachers		559*	17			2011	Rahman *et al.*[16]
Turkey	Primary school teachers		468*	58.1	36.1	5.8	2008	Unsal *et al.*[21]
Tunisia	Primary and secondary school teachers	402	89.1	29.3			2006	Abdelaziz *et al.*[19]
Yemen	Secondary school teachers		317*	8			2006	Bin Ghouth *et al.*[8]
Kenya	Primary school teachers	910	87.9	7	8.4	84.6	2001	Kwamanga *et al.*[11]
Malaysia	Secondary school teachers	180	180*	40.6			2001	Naing & Ahmad [23]
Japan	Kindergarten, elementary and secondary school teachers	16000	87.5	44.7 Male			2000	Ohida *et al.*[26]
				3.1 Female				
Spain	Primary and secondary school teachers	8000	38.1	29.7			2000	Barrueco *et al.*[20]
Syria	Primary and secondary school teachers		90	52.1 Male			2000	Maziak *et al.*[22]
				12.3 Female				
Malaysia	Secondary school teachers	5112	63	20			1994	Bin Yaacob & Bin Harum [18]
Bahrain	Primary and secondary school teachers	1284	89	7	3.1	89.9		Alnasir [14]

^a^Response rate of the study (*Total number of respondents listed as the response rate was not provided), ^b^Publication year.

The low smoking prevalence among Botswana teachers can be, perhaps, attributed to a general non acceptance of smoking in the country, generally. The prevalence of any tobacco smoking and cigarette smoking in Botswana as of 2011 was 17% and 13% respectively [24]. Low prevalence of smoking in Botswana could also be attributed to tobacco control measures that have been put in place in the country. The Government of Botswana long recognised and accepted the need to sensitize its population to the harmful effects of tobacco. Botswana is one of the first African countries to become signatories to the Framework Convention on Tobacco Control (FCTC). Botswana signed FCTC in June 2003 and ratified in 2005. Prior to this development, Botswana had enacted her first tobacco control legislation, the Control of Smoking Act (CSA) in 1992. The main focus of the act is on controlling Environmental Tobacco Smoke in enclosed public and workplace, educational institutions and hospitals as well as to ban tobacco advertising. To date, the country has by far successfully implemented several key aspects of the FCTC guidelines such as smoke free places, a ban on advertising and promotion of tobacco products, and sale to minors. However, the are no systems in place to check compliance [25].

The results of this study demonstrated that male teachers had a significantly higher prevalence of tobacco smoking than their female colleagues (10.8% vs 0.4%, p < 0.001). Similar results have been found in other studies conducted in Japan where, only 3.1% and 44.7% of female and male teachers respectively, were smokers [26], and in Syria where 12.3% of female and 52.1% male teachers were smokers [22]. In addition, 94% of smoking teachers in Bahrain were male teachers [14]. Comparably, other studies have also reported that smoking was higher among male than female teachers [9,16,27]. Interestingly, the results of studies conducted among primary school teachers in Belgaum City, India [15] and secondary school teachers in Yemen [8], indicated that female teachers in these studies did not smoke. Low prevalence of smoking among female teachers could be because traditionally it is a taboo for women to smoke. It has been suggested that there are few female smokers than males especially in developing countries which could probably be related to social norm that has been long formed in many societies [9].

In this study, cigarette smoking was found to be associated with marital status (p = 0.001). Similar findings were reported by Malay secondary school teachers [9]. School level (p = 0.002) and body mass index (p = 0.027) were also significantly associated with smoking among school teachers in Botswana. However, age, education level, number of children less than six years, length of employment, working hours and number of students taught were not significantly associated with smoking.

Smokers in this study indicated that they have been smoking for periods ranging from a year to 31 years with an average smoking duration of 8.62 years, smoking between one to 20 cigarettes a day. The average number of cigarettes smoked was 5.6 per day. The results also show that 5.3% of teachers in the study were ex-smokers having smoked for one to 27 years with average smoking years of 7.83 years.

Various strengths and limitation were found for this study. Firstly, the study covered seven out of 10 educational regions in Botswana, so the results can be generalised. Secondly, the study helped to find the prevalence of smoking among teachers as they are considered to be students’ role models. A limitation of the study is that the data reflect respondents’ subjective perceptions.

## Conclusion

Prevalence of tobacco smoking among Botswana teachers was relatively low. Factors such as gender, school level and body mass index have been associated with smoking. Measures should be put in place to monitor compliance with measures that have been put in place to control tobacco smoking.

## Competing interests

The authors declare they have no competing interests.

## Authors’ contributions

PNE and DRS conceived and designed the study. PNE carried out data collection and analysis. PNE and DRS read and approved the final manuscript.
